# Role of Fiberoptic Bronchoscopy in Patients With Sputum-Negative and Clinico-Radiological Suspicious Pulmonary Tuberculosis

**DOI:** 10.7759/cureus.108651

**Published:** 2026-05-11

**Authors:** Manas Chauda, Naveen Gandhi, Shivani Chaturvedi, Shahzad Husain Arastu, Ashish Dubey, Animesh Dubey, Gaurav Sahu, Gyanprakash Verma, Lalima Gupta

**Affiliations:** 1 Pulmonary Medicine, L.N. Medical College and Research Centre and J.K. Hospital, Bhopal, IND; 2 Community Medicine, Ram Krishna Medical College Hospital and Research Centre, Bhopal, IND

**Keywords:** cartridge based nucleic acid amplification test (cbnaat), chest xray imaging, fiber optic bronchoscopy, fiberoptic bronchoscopy, tuberculosis(tb)

## Abstract

Tuberculosis (TB) remains a major global public health concern, particularly in high-burden countries such as India. Pulmonary TB (PTB) is the most common form of the disease and the principal source of transmission. Although sputum smear microscopy using Ziehl-Neelsen (ZN) staining is widely employed for diagnosis, it has limited sensitivity, especially in patients with sputum smear-negative disease. These patients often present with strong clinical symptoms and radiological findings suggestive of PTB but lack microbiological confirmation, leading to diagnostic delay, inappropriate empirical therapy, and continued transmission. In this context, fiberoptic bronchoscopy (FOB) with bronchoalveolar lavage (BAL) offers an important diagnostic alternative by enabling direct airway evaluation and targeted sample collection. The use of molecular diagnostics, such as the cartridge-based nucleic acid amplification test (CBNAAT) on BAL samples, has further enhanced diagnostic yield.

This cross-sectional observational study was conducted over 18 months at the Department of Pulmonary Medicine at L.N. Medical College and J.K. Hospital, Bhopal. Adult patients (≥18 years) with clinical features suggestive of PTB, such as cough, fever, weight loss, night sweats, or hemoptysis, and radiological findings consistent with PTB, but with at least two sputum samples negative for acid-fast bacilli (AFB) on ZN staining, were included. Patients with prior anti-tubercular therapy within six months, sputum-positive TB, pregnancy, or contraindications to bronchoscopy were excluded. A total of 97 patients were included.

All patients underwent detailed clinical evaluation, chest imaging, and FOB performed under standard aseptic precautions. BAL samples were collected from radiologically involved lung segments and subjected to AFB smear microscopy and CBNAAT. Statistical analysis was performed to assess associations between clinical features, radiological findings, and BAL-CBNAAT results.

The mean age of the study population was 48.98 ± 16.37 years. Almost all patients (95/97; 97.9%) were symptomatic, with cough (88/97; 90.7%) and fever (83/97; 85.6%) being the most common presenting complaints, followed by weight loss (77/97; 79.4%) and night sweats (56/97; 57.7%). Hemoptysis was infrequent (5/97; 5.2%). All patients had radiological findings suggestive of PTB as per the inclusion criteria. BAL-AFB smear was negative in all cases, highlighting the limited sensitivity of smear microscopy in sputum-negative disease. In contrast, BAL-CBNAAT detected Mycobacterium TB in 89 of 97 patients, yielding a diagnostic positivity rate of 91.75%. A statistically significant association was observed between BAL-CBNAAT positivity and clinical symptoms such as cough, fever, weight loss, and night sweats (p < 0.05), while hemoptysis showed no significant correlation.

The study concludes that FOB with BAL, when combined with CBNAAT, has a high diagnostic yield in sputum smear-negative patients with clinico-radiological suspicion of PTB. This approach effectively overcomes the limitations of sputum-based diagnostics, enables early microbiological confirmation, and facilitates timely initiation of appropriate therapy. FOB should be considered a crucial diagnostic tool in the evaluation of sputum smear-negative PTB.

## Introduction

Tuberculosis (TB) continues to be a formidable global health challenge, with an estimated 10.6 million new cases and 1.6 million deaths reported worldwide in 2021, positioning it as a leading cause of infectious disease mortality [1]. Despite concerted global efforts to control TB, high-burden countries, particularly in South Asia and Sub-Saharan Africa, bear a disproportionate burden, with India alone accounting for approximately 26% of global cases [2]. Pulmonary TB (PTB), the most common form of the disease, is responsible for the majority of transmission, making early and accurate diagnosis critical to reducing morbidity, mortality, and community spread [3]. However, diagnosing PTB remains complex, especially in cases where conventional diagnostic tools fail to provide microbiological confirmation, such as in sputum smear-negative patients with clinico-radiological features suggestive of TB [4].

Sputum smear-negative PTB, characterized by the absence of acid-fast bacilli (AFB) on Ziehl-Neelsen (ZN) staining of sputum samples, poses a significant diagnostic dilemma. These cases are prevalent in early-stage disease, paucibacillary TB, immunocompromised patients (e.g., those with HIV co-infection), and atypical presentations [5]. Clinically, these patients often present with symptoms such as persistent cough, fever, night sweats, and weight loss, while radiological findings may include upper lobe infiltrates, cavitation, tree-in-bud patterns, or miliary nodules [6]. However, such clinico-radiological features lack specificity, as they can overlap with other pulmonary conditions, including bacterial pneumonia, lung cancer, nontuberculous mycobacterial infections, and fungal diseases [7]. This diagnostic uncertainty frequently leads to delays in treatment initiation, increasing the risk of disease progression, transmission, and poorer clinical outcomes [8].

The limitations of conventional diagnostics exacerbate the challenge of managing sputum smear-negative TB. Sputum smear microscopy, a cornerstone of TB diagnosis in resource-limited settings, has a sensitivity of only 50-60% in PTB and is particularly insensitive in smear-negative cases [9]. While sputum culture remains the gold standard for microbiological confirmation, its turnaround time of two to six weeks limits its utility in urgent clinical decision-making [10]. Molecular tests, such as GeneXpert MTB/RIF, have revolutionized TB diagnosis by offering rapid detection of Mycobacterium TB and rifampicin resistance; however, the sensitivity of sputum-based GeneXpert in smear-negative PTB remains suboptimal, ranging from 60-70% [11]. These diagnostic gaps highlight the need for alternative approaches to improve the yield of microbiological and histopathological confirmation in this patient population.

Fiberoptic bronchoscopy (FOB) has emerged as a pivotal diagnostic tool in the evaluation of sputum smear-negative, clinico-radiologically suspicious PTB. This minimally invasive procedure enables direct visualization of the tracheobronchial tree and facilitates the collection of high-quality samples, including bronchoalveolar lavage (BAL), bronchial washings, and endobronchial or transbronchial biopsies [12]. BAL samples, in particular, have shown a higher diagnostic yield compared to sputum, with studies reporting culture positivity rates of 30-90% in smear-negative TB cases [13,14]. Additionally, GeneXpert testing on BAL fluid has demonstrated improved sensitivity over sputum-based testing, further enhancing the utility of bronchoscopy [15]. Beyond microbiological confirmation, bronchoscopy allows histopathological examination of biopsy specimens, which may reveal granulomatous inflammation or caseation necrosis suggestive of TB, aiding in diagnosis when cultures are negative [16]. Importantly, bronchoscopy also plays a critical role in excluding alternative diagnoses, such as malignancy or fungal infections, which is essential for guiding appropriate therapy [17].

Despite its diagnostic potential, the use of FOB in TB-endemic settings is limited by several factors. The procedure is invasive, requiring specialized equipment, trained personnel, and infection control measures to prevent nosocomial transmission of TB [18]. In resource-constrained settings, access to bronchoscopy services is often restricted to tertiary care centers, posing logistical and financial barriers for patients [19]. Furthermore, the procedure carries risks, albeit low, including bleeding, pneumothorax, and post-procedural infections, which must be weighed against its diagnostic benefits [20]. These challenges underscore the need to evaluate the role of bronchoscopy systematically, particularly in high-TB-burden regions where sputum smear-negative cases are prevalent.

The significance of studying FOB in this context lies in its potential to bridge the diagnostic gap for sputum smear-negative, clinico-radiologically suspicious PTB. By improving diagnostic accuracy, bronchoscopy can facilitate timely treatment, reduce unnecessary empirical therapy, and minimize the risk of drug-resistant TB due to inappropriate management [21]. Moreover, understanding the clinical, microbiological, and radiological predictors of positive bronchoscopy findings can help optimize patient selection and resource utilization, making the procedure more cost-effective in resource-limited settings [22]. This study seeks to contribute to the growing body of evidence on the utility of FOB, addressing a critical need in the effective diagnosis and control of PTB in high-burden regions.

The primary objective of this study was to determine the diagnostic yield of BAL-cartridge-based nucleic acid amplification test (CBNAAT) obtained via FOB in sputum smear-negative, clinico-radiologically suspected PTB patients. Secondary objectives included assessing the association between clinical symptoms and BAL-CBNAAT positivity.

## Materials and methods

This cross-sectional observational study was conducted at the Department of Pulmonary Medicine at L.N. Medical College and J.K. Hospital, Bhopal, over a period of 18 months, after obtaining institutional ethical approval (LNMC&RC/Dean/2024/Ethics/320). Adult patients (≥18 years) with clinical symptoms and radiological findings suggestive of PTB and negative sputum smears for AFB were included. Patients with prior anti-tubercular therapy within six months, contraindications to bronchoscopy, pregnancy, or sputum-positive disease were excluded.

A total of 97 patients meeting the inclusion criteria were enrolled during the study period. The sample size was determined by the number of eligible patients presenting to the center over the study duration.

All participants underwent detailed clinical evaluation, sputum examination, radiological assessment, and routine laboratory investigations. FOB was performed under standard institutional protocols by experienced pulmonologists, and BAL samples were collected from radiologically involved segments under aseptic precautions.

BAL samples were analyzed using ZN staining and CBNAAT using the GeneXpert MTB/RIF platform, which also provides information on rifampicin resistance. Data on demographic, clinical, radiological, bronchoscopic, and microbiological parameters were recorded in a structured proforma. Diagnostic yield was defined as the proportion of BAL-CBNAAT-positive cases among the total number of patients evaluated.

Statistical analysis was performed using standard software. Continuous variables were expressed as mean ± standard deviation, and categorical variables as frequencies and percentages. Associations were assessed using the chi-square test, with a p-value < 0.05 considered statistically significant.

## Results

A total of 97 patients were included in the study. The mean age of the study population was 48.98 ± 16.37 years. Table 1 shows the BAL-CBNAAT positivity rate. BAL-CBNAAT detected Mycobacterium TB in 89 out of 97 patients (91.75%).

**Table 1:** BAL-CBNAAT positivity rate BAL: bronchoalveolar lavage; CBNAAT: cartridge-based nucleic acid amplification test

Variable	Value
Total patients	97
CBNAAT positive	89
CBNAAT negative	8
Diagnostic yield	91.75%

Table 2 shows that the majority of patients (95/97; 97.9%) exhibited at least one symptom suggestive of PTB. Fever (83/97; 85.6%) and cough (88/97; 90.7%) were the most frequently reported symptoms, followed by weight loss (77/97; 79.4%), night sweats (56/97; 57.7%), and hemoptysis (5/97; 5.2%). This pattern highlights the predominance of constitutional and respiratory symptoms among sputum-negative TB suspects.

**Table 2:** Distribution of participants for symptomatology TB: tuberculosis

Variable	Category	Frequency	Percentage
Symptom Presentation	Present	95	97.9%
	Absent	2	2.1%
Cough Presentation	Present	88	90.7%
	Absent	9	9.3%
Fever Presentation	Present	83	85.6%
	Absent	14	14.4%
Night Sweat	Present	56	57.7%
	Absent	41	42.3%
Hemoptysis	Present	5	5.2%
	Absent	92	94.8%
Weight Loss	Present	77	79.4%
	Absent	20	20.6%
Previous TB Test	Conducted	72	74.2%
	Not Conducted	25	25.8%

In Table 3 (n = 97), a statistically significant association was observed between overall symptom presence and BAL-CBNAAT positivity (p < 0.001, df = 1; effect size = 0.484), indicating a moderate association. Among symptomatic patients, 89 (91.75%) were CBNAAT positive, whereas among asymptomatic individuals, zero (0%) tested positive. The mean age of patients was 48.98 ± 16.37 years

**Table 3:** Combined symptom association NA: not applicable; CBNAAT: cartridge-based nucleic acid amplification test; AFB: acid-fast bacilli

Variable	Category	CBNAAT		P-value	Likelihood Ratio	df	Effect Size
		Positive	Negative				
Symptoms	Present	89 (91.75%)	6 (6.18%)	<0.001	<0.001	1	0.484
	Absent	0 (0.00%)	2 (2.06%)				
Cough	Present	83 (85.56%)	5 (5.15%)	0.002	0.020	1	0.292
	Absent	6 (6.18%)	3 (3.09%)				
Weight Loss	Present	74(76.28%)	3 (3.09%)	<0.001	0.024	1	0.310
	Absent	15 (15.46%)	5 (5.15%)				
Fever	Present	79 (81.44%)	4 (4.12%)	0.003	0.011	1	0.303
	Absent	10 (10.30%)	4 (4.12%)				
Night Sweat	Present	55 (56.70%)	1 (1.03%)	<0.001	0.007	1	0.275
	Absent	34 (35.05%)	7 (7.21%)				
Radiological Finding	Suggestive	89 (83.91%)	8 (16.08%)	N A	NA	NA	NA
	Negative	0 (0%)	0 (0%)				
AFB Finding	Negative	89 (83.91%)	8 (16.08%)	N A	NA	NA	NA
	Positive	0 (0%)	0 (0%)				

Cough showed a statistically significant association with CBNAAT positivity (p = 0.002, df = 1; effect size = 0.292). Among patients presenting with cough, 83 (85.56%) were CBNAAT positive, compared to six (6.18%) among those without cough. Weight loss demonstrated a significant association with CBNAAT positivity (p < 0.001, df = 1; effect size = 0.310). Among patients reporting weight loss, 74 (76.28%) were CBNAAT positive, whereas 15 (15.46%) among those without weight loss tested positive. Fever was significantly associated with CBNAAT positivity (p = 0.003, df = 1; effect size = 0.303). Among febrile patients, 79 (81.44%) were CBNAAT positive, compared to 10 (10.30%) among those without fever. Night sweats also showed a statistically significant association with CBNAAT positivity (p < 0.001, df = 1; effect size = 0.275). Among patients with night sweats, 55 (56.70%) were CBNAAT positive, whereas 34 (35.05%) among those without night sweats tested positive. All patients (97/97; 100%) had radiological findings suggestive of PTB as per the inclusion criteria; therefore, statistical association with CBNAAT positivity could not be assessed. Similarly, BAL AFB smear was negative in all patients (97/97; 100%), and correlation with CBNAAT positivity was not feasible.

## Discussion

The present study evaluated the diagnostic utility of FOB with BAL, particularly BAL-based CBNAAT, in patients with sputum smear-negative PTB who had strong clinical and radiological suspicion of disease. The findings of this study are discussed below in comparison with previously published studies, highlighting areas of concordance and contradiction.

In the present study, the mean age of patients was 48.98 ± 16.37 years, with the majority belonging to the middle-aged and elderly population. This finding is in accordance with studies by Kalawat U et al., Luhadia A et al., and Sanjeevaiah S et al., where most sputum-negative PTB patients were middle-aged adults [17,18,22]. Mishra MM et al. also reported a higher burden of disease in the 45-60-year age group, closely mirroring the age distribution seen in the current study [20].

However, some studies, such as the one reported by Johar et al., show a younger predominance (20-40 years) [21]. This difference may be attributable to regional demographic variation, referral patterns, and inclusion of outpatient populations, whereas the present study was conducted in a tertiary care setting where older patients with persistent or unresolved symptoms are more frequently referred.

Gender distribution in the present study showed a near-equal involvement of males and females, with a slight female predominance. This finding contrasts with most studies in the literature, including those by Sanjeevaiah S et al., Kumar KR et al., and Ali GA et al., which demonstrated a clear male predominance [19,22,23]. The relatively higher proportion of females in the current study may reflect improved health-seeking behavior among women or sociocultural differences in the study population. Nonetheless, the absence of marked gender disparity supports the understanding that sputum-negative PTB affects both sexes significantly.

Cough and fever were the most common symptoms in the present study, followed by weight loss and night sweats. These findings are consistent with observations by Luhadia A et al., Mishra MM et al., and Mukharya AD et al., who reported cough as the predominant presenting symptom in sputum-negative PTB patients [18,20,24]. Constitutional symptoms such as fever and weight loss were also commonly observed in these studies, reinforcing their diagnostic relevance.

The low prevalence of hemoptysis in the present study is in agreement with findings by Mishra MM et al. and Mukharya AD et al., where hemoptysis was reported infrequently [20,24]. This contrasts with smear-positive disease, where hemoptysis is more common, and supports the concept that sputum-negative PTB is often paucibacillary and less likely to present with cavitary lesions [20].

The strong statistical association between clinical symptoms and BAL-CBNAAT positivity observed in this study aligns with the conclusions of Johar A et al., who demonstrated a significant association between clinical features and microbiological positivity on BAL [21]. This reinforces the role of symptom-based clinical suspicion in guiding invasive diagnostic procedures.

All patients in the present study had radiological findings suggestive of PTB, as per the inclusion criteria. This is similar to previous studies, where radiological suspicion formed the basis for subjecting patients to bronchoscopy [18,21,25]. Upper zone involvement and infiltrative lesions were commonly reported in these studies, although detailed zonal analysis was not the focus of the current work.

Unlike Johar A et al., who were able to demonstrate statistically significant associations between specific radiological patterns (cavitation, nodules, tree-in-bud) and BAL positivity, the present study could not perform such an association due to the absence of radiologically negative controls and also due to the inclusion of only radiologically positive participants [21]. This represents a methodological difference rather than a contradiction and highlights a limitation of the current study design.

The overall diagnostic yield of BAL-CBNAAT in the present study was 91.75%, which is higher than most previously published studies. Kalawat U et al. reported an overall diagnostic yield of 86.6% using BAL smear and culture, while Luhadia A et al. achieved a yield of 86% when combining BAL, post-bronchoscopy sputum, and biopsy results [17,18]. Similarly, Sanjeevaiah S et al. reported culture positivity in 66.66% of cases, and Quaiser S et al. confirmed diagnosis in 60% of sputum-negative patients [22,26].

The higher yield in the present study may be attributed to the use of CBNAAT, which has superior sensitivity compared to smear microscopy and provides rapid detection in paucibacillary disease. Studies by Kumar KR et al. and Sivaji S et al. demonstrated BAL-CBNAAT positivity rates of 41.6% and 85%, respectively, supporting the enhanced diagnostic value of molecular testing but still reporting lower yields than the current study [19,25].

This apparent discrepancy may reflect stricter inclusion criteria, higher pre-test probability, and tertiary care referral bias in the present study. Therefore, while the findings are in accordance with the literature regarding the superiority of BAL-CBNAAT, the magnitude of yield should be interpreted with caution.

In the present study, BAL AFB smear was negative in all patients, highlighting its limited sensitivity in sputum-negative PTB. This observation is partially in agreement with Mishra MM et al. and Mukharya AD et al., who also reported low BAL AFB positivity rates (5.6% and 10.67%, respectively) [20,24]. However, it contrasts with studies by Sanjeevaiah S et al. and Johar A et al., where BAL AFB positivity exceeded 50% [21,22].

These variations may be due to differences in disease burden, bacillary load, sample processing techniques, and patient selection. The uniformly negative BAL AFB results in the present study emphasize the limited standalone role of smear microscopy and strongly support the preferential use of CBNAAT in sputum-negative cases.

Several studies in the literature, including those by Ali GA et al., Gupta DV et al., and Omer AM et al., demonstrated improved diagnostic yield when BAL was combined with post-bronchoscopy sputum, bronchial brushing, or biopsy [23,27,28]. In contrast, the present study focused exclusively on BAL samples, particularly CBNAAT, without incorporating additional bronchoscopic specimens.

Despite this, the diagnostic yield achieved in the current study was comparable to studies using multiple modalities. This finding supports the efficiency of BAL-CBNAAT as a single, high-yield diagnostic test, especially in resource-limited settings where extensive sampling may not be feasible [29].

Overall, the findings of the present study are largely in accordance with existing literature in demonstrating that FOB significantly enhances diagnostic confirmation in sputum smear-negative PTB. The study further reinforces the growing body of evidence favoring CBNAAT over conventional smear microscopy.

Where differences exist, particularly in diagnostic yield and BAL AFB smear positivity, they may be explained by methodological variations rather than true contradiction. The present study contributes to the existing literature by demonstrating an exceptionally high yield of BAL-CBNAAT in a carefully selected cohort, thereby strengthening the argument for early bronchoscopic evaluation in sputum-negative, clinically suspected PTB patients.

The study does come with certain limitations. First, it was conducted at a single tertiary care center, which may limit the generalizability of the findings to broader populations. Second, the sample size was relatively small. Third, all patients included had radiological findings suggestive of PTB, which limited the ability to assess the association between radiological patterns and CBNAAT positivity. Fourth, only BAL samples were evaluated, and additional bronchoscopic modalities such as biopsy or bronchial brushing were not included. Finally, as a cross-sectional study, causal relationships could not be established. Additionally, mycobacterial culture was not performed in this study, which may have limited comprehensive microbiological confirmation and comparison with CBNAAT results.

## Conclusions

FOB with BAL-CBNAAT demonstrates a high diagnostic yield in sputum smear-negative, clinico-radiologically suspected PTB patients. It offers a useful approach for microbiological confirmation in clinically challenging cases where sputum-based diagnostics are inconclusive. However, larger studies with microbiological confirmation are required to further validate these findings.
